# Increased Blood Pressure Variability Is Associated with Worse Neurologic Outcome in Acute Anterior Circulation Ischemic Stroke

**DOI:** 10.1155/2016/7670161

**Published:** 2016-11-15

**Authors:** Adam de Havenon, Alicia Bennett, Gregory J. Stoddard, Gordon Smith, Haimei Wang, Jana Wold, Lee Chung, David L. Tirschwell, Jennifer J. Majersik

**Affiliations:** ^1^Department of Neurology, University of Utah, Salt Lake City, UT, USA; ^2^Department of Internal Medicine, University of Utah, Salt Lake City, UT, USA; ^3^Department of Neurology, University of Washington, Seattle, WA, USA

## Abstract

*Background*. Although research suggests that blood pressure variability (BPV) is detrimental in the weeks to months after acute ischemic stroke, it has not been adequately studied in the acute setting.* Methods*. We reviewed acute ischemic stroke patients from 2007 to 2014 with anterior circulation stroke. Mean blood pressure and three BPV indices (standard deviation, coefficient of variation, and successive variation) for the intervals 0–24, 0–72, and 0–120 hours after admission were correlated with follow-up modified Rankin Scale (mRS) in ordinal logistic regression models. The correlation between BPV and mRS was further analyzed by terciles of clinically informative stratifications.* Results*. Two hundred and fifteen patients met inclusion criteria. At all time intervals, increased systolic BPV was associated with higher mRS, but the relationship was not significant for diastolic BPV or mean blood pressure. This association was strongest in patients with proximal stroke parent artery vessel occlusion and lower mean blood pressure.* Conclusion*. Increased early systolic BPV is associated with worse neurologic outcome after ischemic stroke. This association is strongest in patients with lower mean blood pressure and proximal vessel occlusion, often despite endovascular or thrombolytic therapy. This hypothesis-generating dataset suggests potential benefit for interventions aimed at reducing BPV in this patient population.

## 1. Introduction

Data from acute ischemic stroke clinical trials suggests a U-shaped relationship between blood pressure and neurologic outcome, with extremes of both hypo- and hypertension associated with worse outcome [1–3]. Over 60% of ischemic stroke patients have a transient acute hypertensive response, which is theorized to be the result of different mechanisms including stroke-specific augmentation of cerebral perfusion through collateral blood flow [4]. Numerous clinical trials have been conducted to determine if pharmacologically lowering blood pressure in the acute period after ischemic stroke is beneficial. The results have been persistently negative or neutral [5]. Post hoc analysis and other observational datasets suggest increased blood pressure variability (BPV), independent of mean blood pressure, is harmful after ischemic and hemorrhagic stroke [2, 6–10]. The first systematic meta-analysis of BPV and stroke was recently published [11], which supported this association, but there was significant heterogeneity in patient selection, concurrent clinical trial interventional treatment, frequency of blood pressure measurement, definition of neurologic outcome, and the statistical measures of variability.

Two prior studies of BPV after ischemic stroke were restricted to proximal anterior circulation occlusion and both showed a strong association between increased BPV and worse neurologic outcome [12, 13]. However, these studies had small sample sizes, only collected blood pressure measurements for 24 and 48 hours after admission, and did not fully address the contribution of mean blood pressure to outcome. Based on this data and the negative clinical trials of blood pressure reduction after stroke, we hypothesized that increased BPV would be associated with worse neurologic outcome, particularly in patients with proximal vessel occlusions and lower mean blood pressure. We addressed this hypothesis by studying a retrospective cohort of anterior circulation ischemic stroke.

## 2. Methods

Subjects were retrospectively identified by searching the electronic medical record of a comprehensive stroke center for patients with anterior circulation ischemic stroke diagnosed by a neurologist (defined as within the vascular territory of the internal carotid artery) and an mRS between 30 and 365 days after stroke onset, using the mRS closest to 90 days after stroke onset. We also recorded mRS at hospital discharge and if it was 0 (no symptoms) or 6 (death), we carried it forward as a follow-up mRS because of the terminal value. Patients were excluded if there was insufficient radiographic data to determine persistent proximal vessel occlusion (PPVO), which was defined as persistent occlusion of the stroke-ipsilateral internal carotid artery or M1 segment of the middle cerebral artery on MR, CT, or digital subtraction angiography after endovascular intervention or up to 72 hours after admission or thrombolytic therapy. Patients who underwent endovascular intervention for proximal occlusion and achieved a postprocedural thrombolysis in cerebral infarction score of >0 were not considered to have PPVO. Patients with proximal occlusion on admission who did not undergo endovascular intervention were included in the cohort if they had follow-up angiographic imaging within 72 hours of hospital admission to verify outcome of proximal vessel occlusion, as some patients spontaneously recanalize or do so after IV tPA [14].

**Figure 1:**
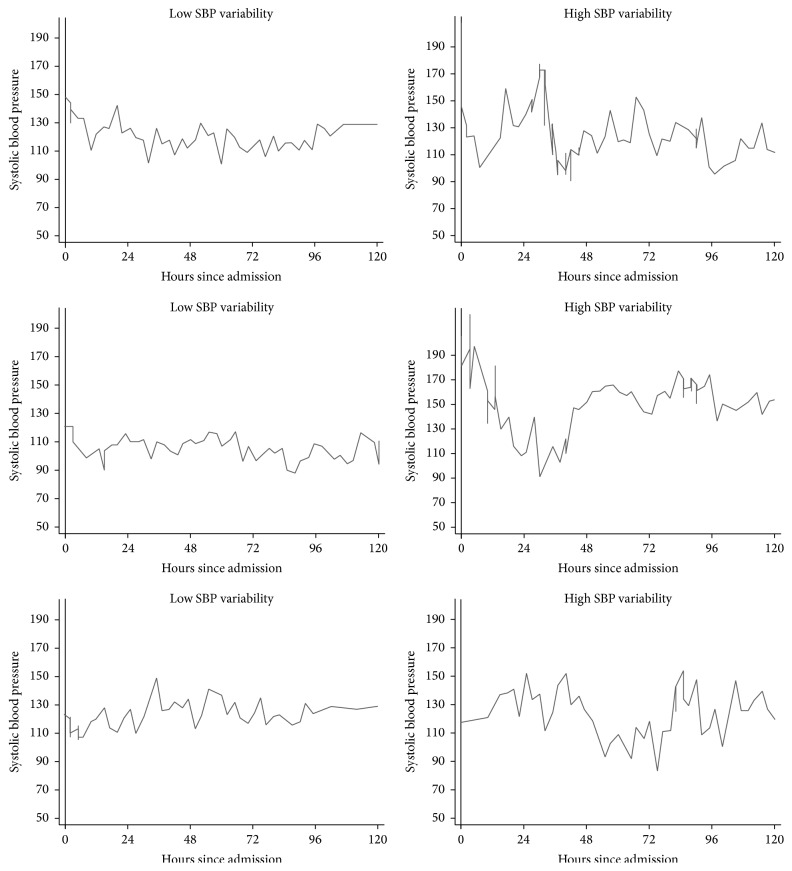
Systolic blood pressure plotted against hours since hospital admission in 6 representative patients, showing high and low variability.

Additional information was obtained from the electronic medical record, including medical comorbidities and all hemodynamic data from the inpatient admission. We defined endovascular intervention as mechanical and aspiration thrombectomy or intra-arterial tPA and symptomatic intracerebral hemorrhage (sICH) according to the European Cooperative Acute Stroke Study 2 definition [15]. We analyzed BPV over 3 time intervals: 0–24, 0–72, and 0–120 hours after admission (Figure 1). Over 80% of patients had hemodynamic data starting within 6 hours of stroke onset and the remainder had it within 24 hours. The number of patients in sequential time intervals decreased secondary to discharge or death. We represented BPV as standard deviation (SD), coefficient of variation (CV), and successive variation (SV) [8]. SD is a measure of the average distance the blood pressure values are from the mean and is heavily influenced by outliers. CV is the ratio of SD to mean blood pressure and thus represents blood pressure dispersion independent of mmHg. SV takes into account temporal changes in blood pressure by comparing measurements between nearest neighbors in time rather than to the overall mean (as the SD does) and better represents BPV when patients have alternating patterns of increasing and decreasing blood pressure [9]. In keeping with recommendations from the recent meta-analysis on BPV, we reported odds ratios (ORs) and 95% confidence intervals (CIs) per 10 mmHg blood pressure increment [11]. We assessed the direct relationship between BPV indices and mRS using Spearman's rank correlation coefficient (Spearman's rho).

Historically, the primary outcome in stroke trials has been a binary dichotomization of mRS, but more recently stroke trialists have preferred statistical methodologies that measure shift in mRS, which are particularly beneficial when the effect of the intervention or clinical factor is spread across the entire range of ordinal values [16–18]. Acknowledging this, we primarily fitted ordinal logistic regression models to the outcome of a one-point shift in mRS with BPV indices (SD, CV, and SV) as the predictors. Clinically, we suspected that the effect of the BPV indices would be modified by stroke parent vessel status and blood pressure mean, so the models were also stratified by these factors. For adjusted analyses, we included variables that remained in the model with *p* < 0.10 in the strata with the least number of patients, a liberal significance criterion that increases protection against residual confounding. We verified the proportional hazards assumption of the ordinal logistic regression models using the Brant test [19].

For all stratifications, the ordinal regression models are calculated separately for each stratum. To investigate the effect of BPV on stroke parent vessel status, we made two stratifications: patients with or without proximal vessel occlusion at hospital admission and patients with or without persistent proximal vessel occlusion (PPVO). We examined the effect of BPV on patients with different mean SBP by stratifying the cohort into terciles of mean SBP and performing an unadjusted and adjusted ordinal logistic regression with the outcome of a one-point shift in the follow-up mRS. An attempt was made to stratify patients based on antihypertensive treatment, which can affect BPV, but the vast heterogeneity in treatment modalities, frequencies, and dosages made such a stratification impossible.

## 3. Results

Two hundred thirty-three patients met initial inclusion criteria, but 18 were subsequently excluded due to insufficient data for PPVO determination, leaving 215 in the final cohort. Patient demographics are shown in Table 1. The median number of blood pressure readings per patient was 15 (IQR 11–22) during the time interval 0–24 hours, 26 (IQR 22–33) during 0–72 hours, and 34 (IQR 28–39) during 0–120 hours. Approximately half of patients were given intravenous tPA or had endovascular intervention, and 34% (72/215) had both. There were a large number of proximal vessel occlusions at hospital admission (67%, 144/215), but the number of PPVOs fell to 25% (54/215) after endovascular intervention and tPA. The median NIH stroke scale (NIHSS) was 14 (IQR 8–20) and mRS was 3 (IQR 1–6), reflecting the higher stroke severity associated with proximal vessel occlusion.

The mean ± SD of SBP, DBP, and BPV indices is shown for the corresponding time periods in Table 2. The Spearman rank correlations between BPV and mRS showed a significant positive association at all time points between mRS and indices of systolic BPV (SD, CV, and SV), but the relationship was inconsistent for diastolic BPV, mean SBP, and mean DBP, all in keeping with prior reports (Table 2) [11]. In the unadjusted and adjusted logistic regression models, SBP CV had the highest ORs for a one-point increase in mRS or death (Table 3). Based on these results, we chose SBP CV as the primary predictor in the stratified regression analyses. When the cohort was stratified by proximal vessel occlusion at hospital admission (PVO) and PPVO, the odds of a one-point increase in follow-up mRS were higher in patients with PVO (OR 3.13–7.25, *p* < 0.05) and PPVO (4.32–18.1, *p* < 0.05) than patients without proximal vessel occlusion (Table 4). This association persisted in multivariable models adjusting for admission NIHSS, patient age, admission glucose, premorbid mRS, endovascular therapy, and IV tPA administration (OR 2.75–48.9, *p* < 0.05). The wide confidence intervals for the PPVO stratification confer some uncertainty, but the results were consistent with the PVO stratification, suggesting the effect is valid.

In the third stratification, using mean SBP terciles, the stratum with the lowest mean SBP was the only one to demonstrate an association between increased SBP CV and a one-point increase in mRS (OR 3.76–10.5, *p* < 0.05) (Table 5), which remained after adjusting for admission NIHSS, patient age, admission glucose, PPVO, and premorbid mRS (OR 4.05–8.77, *p* < 0.05). The terciles of mean SBP were not independently associated with follow-up mRS (rho = −0.05, *p* > 0.05), confirming that this stratification did not confound the interaction between SBP CV and mRS.

## 4. Discussion

Our analysis indicates that increased systolic BPV is associated with worse neurologic outcome after acute anterior circulation ischemic stroke and that patients with proximal vessel occlusion and lower mean SBP may be particularly susceptible to this detrimental effect. Similar to prior research, we find that systolic BPV is a better predictor than diastolic BPV, and both are better than mean blood pressure [11]. One other study specifically compared SBP SD, CV, and SV in ischemic stroke patients and found SBP CV to be superior but only included patients who had received IV tPA [9]. Based on our data showing SBP CV to be the best predictor of neurologic outcome among BPV indices, it should be included in future analyses of BPV.

Under normal circumstances, dynamic autoregulation of the cerebrovascular bed maintains a relatively constant cerebral blood flow across a wide range of blood pressure measurements [20]. However, after moderate to severe ischemic stroke, autoregulation is lost in the area of the stroke core and ischemic penumbra [21, 22]. Patients with PVO and PPVO may be susceptible to the detrimental effects of increased systolic BPV given their greater stroke severity. Furthermore, patients with lower mean SBP may be particularly vulnerable to hypoxic conditions during periods of increased BPV because of impaired autoregulation combined with inherently lower cerebral perfusion pressure. This may explain why the majority of antihypertensive trials in patients with ischemic stroke resulted in no benefit or worse outcomes in the treated groups [5]. It is also concerning that the recommended medication for lowering blood pressure in acute ischemic stroke is the *β* blocker labetalol, which can increase BPV compared to other antihypertensives such as calcium-channel blockers [23, 24].

These results are particularly significant because they suggest that patients with PPVO, many of whom have failed endovascular therapy or IV tPA, may benefit from subsequent interventions designed to reduce BPV. However, the therapeutic potential of agents which may reduce BPV is poorly explored. Although the effect of vasopressor medications on BPV has not been well studied in patients with ischemic stroke, the infusion of phenylephrine into healthy volunteers produces a dose-dependent linear increase in blood pressure and once the vasopressor reaches a steady state, the natural diurnal variations of blood pressure are reduced [25, 26]. The only randomized controlled trial of vasopressor administration in ischemic stroke patients showed a significant improvement in neurologic outcome with no adverse events [27], but there was no difference in mean blood pressure between patients receiving the vasopressor and controls. This raises the question of whether the observed benefit was due to a reduction in BPV, which was not reported [27].

Our study has advantages over existing datasets. We restricted our analysis to anterior circulation stroke, did not have concurrent interventions from clinical trials, and have data on the status of proximal vessel occlusions after admission and treatment and a large number of blood pressure measurements. The most significant limitation of our study is its retrospective nature, which yielded nonuniform time intervals between blood pressure measurements, time from stroke onset to first blood pressure measurement, and hospital discharge to clinical follow-up. Nonetheless, with the large number of blood pressure measurements per patient, our data remain informative because of the association of increased BPV and worse neurologic outcome in patients with lower mean SBP and proximal vessel occlusions, which is hypothesis-generating.

We were not able to adjust for antihypertensive treatment, as it would be impossible to account for the heterogeneity in how patients were treated. A potential confounder for the blood pressure stratification is if patients with lower mean SBP had additional medical complications causing their blood pressure to drop, such as systemic infection. While we are unable to control for these unknown clinical factors, the follow-up mRS did not correlate with the stratifications, suggesting neurologic outcome was not different based on stratification alone. Finally, an inclusion bias may have been introduced by the small number of patients (18/233, 8%) who initially met inclusion criteria but were excluded because they did not have sufficient radiographic data to determine PPVO.

## 5. Conclusion

Systolic BPV, particularly SBP CV, is associated with neurologic outcome after anterior circulation ischemic stroke and perhaps more so in patients with proximal vessel occlusion and lower mean systolic blood pressure. Our data further imply that ischemic stroke patients with proximal anterior circulation occlusion who fail or are not candidates for endovascular intervention or IV tPA may specifically benefit from therapies aimed at reducing BPV in the first 5 days following stroke onset. Prior clinical trials of antihypertensive medications known to reduce BPV, primarily calcium-channel blockers, have not shown benefit for acute ischemic stroke patients [5, 23, 28]. A potential approach to lowering BPV in acute ischemic stroke patients may be vasopressor medications [25, 26], which have shown benefit in patients with moderate-severe ischemic stroke [29].

## Figures and Tables

**Table 1:** Baseline demographics, imaging variables, laboratory values, and stroke risk factors.

Variable	All patients (*n* = 215)
Age (mean ± SD)	62 ± 17
Male, *n* (%)	112 (52.1)
Caucasian, *n* (%)	194 (90.2)
Premorbid mRS (median, IQR)	0 (0-1)
Admission NIHSS (median, IQR)	14 (8–20)
Follow-up mRS (median, IQR)	3 (1–6)
Days from stroke onset to follow-up mRS (mean ± SD)	122 ± 85
Dead at follow-up, *n* (%)	52 (24.2)
Good neurologic outcome (mRS 0-1), *n* (%)	66 (30.7)
Hypertension, *n* (%)	120 (55.8)
Hyperlipidemia, *n* (%)	83 (38.6)
Atrial fibrillation, *n* (%)	66 (30.7)
Diabetes mellitus, *n* (%)	44 (20.5)
Congestive heart failure, *n* (%)	33 (15.4)
Current cigarette smoking, *n* (%)	48 (22.3)
Admission glucose level (mean ± SD)	129 ± 47
Admission blood urea nitrogen level (mean ± SD)	17.8 ± 8.3
Admission international normalized ratio (mean ± SD)	1.2 ± 0.3
IV tPA administered, *n* (%)	105 (48.8)
Endovascular therapy, *n* (%)	131 (60.9)
Symptomatic intracerebral hemorrhage, *n* (%)	27 (12.6)

**Table 2:** Spearman rank correlation coefficient for blood pressure indices and mRS and mean ± SD values.

Variable	Mean ± SD	Spearman rho	*p* value
0–24 hours (*n* = 215 patients, 4,714 vital sign readings)			

SBP SD	13.9 ± 6.5	0.22	0.002
DBP SD	10.8 ± 4.4	0.15	0.03
SBP CV	10.5 ± 4.6	0.18	0.01
DBP CV	15.2 ± 6.0	0.14	0.04
SBP SV	15.3 ± 7.2	0.22	<0.001
DBP SV	12.3 ± 5.5	0.04	0.56
Mean SBP	132.2 ± 18.5	0.14	0.04
Mean DBP	72.2 ± 11.0	−0.07	0.33

0–72 hours (*n* = 202 patients, 9,539 vital sign readings)			

SBP SD	14.7 ± 5.8	0.23	0.002
DBP SD	11.1 ± 3.8	0.09	0.19
SBP CV	11.2 ± 4.3	0.20	0.01
DBP CV	16.1 ± 5.7	0.12	0.09
SBP SV	14.7 ± 5.5	0.23	<0.001
DBP SV	11.9 ± 4.8	−0.01	0.90
Mean SBP	131.0 ± 18.1	0.12	0.09
Mean DBP	70.0 ± 10.6	−0.07	0.30

0–120 hours (*n* = 186 patients, 12,972 vital sign readings)			

SBP SD	15.0 ± 5.5	0.25	0.001
DBP SD	11.1 ± 3.5	0.10	0.16
SBP CV	11.5 ± 4.2	0.22	<0.001
DBP CV	16.3 ± 5.6	0.13	0.08
SBP SV	14.7 ± 5.5	0.25	0.002
DBP SV	11.8 ± 4.5	0.00	0.99
Mean SBP	131.3 ± 18.3	0.10	0.20
Mean DBP	69.5 ± 10.4	−0.07	0.35

**Table 3:** Ordinal logistic regression models with predictor variables of systolic BPV and mean SBP fitted to the outcomes; (a) columns 2–4: one-point shift in mRS, (b) columns 5–7: bad outcome (mRS 3–6), and (c) columns 8–10: death.

Variable	OR for 1-point mRS shift	95% CI	*p* value	Adjusted OR for 1-point mRS shift^*∗*^	95% CI	*p* value	OR for death at follow-up	95% CI	*p* value
0–24 hours (*n* = 215)									

SBP CV	2.32	1.35–4.00	0.002	2.06	1.09–3.92	0.03	2.97	1.54–5.74	0.001
SBP SD	1.99	1.34–2.96	0.001	1.61	1.02–2.55	0.04	2.29	1.42–3.69	0.001
SBP SV	1.83	1.32–2.54	<0.001	1.83	1.22–2.74	0.01	1.94	1.33–2.85	0.001
Mean SBP	1.02	1.00–1.03	0.02	1.00	0.99–1.02	1.00	1.01	0.99–1.03	0.09

0–72 hours (*n* = 202)									

SBP CV	3.38	1.73–6.59	<0.001	2.32	1.03–5.21	0.04	5.62	2.40–13.1	<0.001
SBP SD	2.56	1.55–4.23	<0.001	1.70	0.95–3.04	0.08	3.32	1.80–6.13	<0.001
SBP SV	2.45	1.48–4.07	0.001	2.18	1.20–3.96	0.01	2.72	1.49–4.95	0.001
Mean SBP	1.01	1.00–1.03	0.08	1.00	0.99–1.02	0.72	1.01	0.99–1.03	0.29

0–120 hours (*n* = 186)									

SBP CV	4.33	1.94–9.69	<0.001	3.16	1.25–7.94	0.02	8.79	2.92–26.5	<0.001
SBP SD	3.07	1.72–5.49	<0.001	1.98	1.05–3.74	0.04	4.13	1.95–8.74	<0.001
SBP SV	2.88	1.57–5.29	0.001	2.32	1.20–4.49	0.01	2.81	1.35–5.87	0.01
Mean SBP	1.01	0.99–1.02	0.20	1.00	0.98–1.02	0.96	1.00	0.98–1.03	0.65

^*∗*^Adjusted for admission NIHSS, patient age, history of atrial fibrillation, history of diabetes mellitus, endovascular therapy, IV tPA administration, and premorbid mRS.

**Table 4:** Ordinal logistic regression models fitted to a one-point shift in mRS, stratified by proximal vessel occlusion at hospital admission and persistent proximal vessel occlusion, with predictor variables of SBP CV and SBP CV in a multivariable model.

Variable	OR for 1-point mRS shift	95% CI	*p* value	OR for 1-point mRS shift	95% CI	*p* value
0–24 hours (*n* = 215)						

Cohort	Proximal vessel occlusion at hospital admission (*n* = 144)			No proximal vessel occlusion at hospital admission (*n* = 71)		

SBP CV	3.13	1.53–6.42	0.002	1.02	0.39–2.67	0.98
Adjusted SBP CV model^*∗*^	3.81	1.68–8.65	0.001	0.64	0.22–1.90	0.42

Cohort	Persistent proximal vessel occlusion (*n* = 54)			No persistent proximal vessel occlusion (*n* = 161)		

SBP CV	4.32	1.09–17.1	0.04	2.26	1.16–4.38	0.02
Adjusted SBP CV model^*∗*^	2.75	0.67–11.3	0.02	2.11	1.00–4.44	0.05

0–72 hours (*n* = 202)						

Cohort	Proximal vessel occlusion at hospital admission (*n* = 136)			No proximal vessel occlusion at hospital admission (*n* = 66)		

SBP CV	6.03	2.36–15.4	<0.001	0.94	0.29–3.07	0.92
Adjusted SBP CV model^*∗*^	5.77	1.99–16.7	0.001	0.49	0.14–1.76	0.27

Cohort	Persistent proximal vessel occlusion (*n* = 150)			No persistent proximal vessel occlusion (*n* = 152)		

SBP CV	7.82	1.52–40.2	0.01	2.81	1.21–6.50	0.02
Adjusted SBP CV model^*∗*^	15.5	2.27–106	0.01	1.41	0.56–3.56	0.47

0–120 hours (*n* = 186)						

Cohort	Proximal vessel occlusion at hospital admission (*n* = 125)			No proximal vessel occlusion at hospital admission (*n* = 61)		

SBP CV	7.25	2.38–22.1	<0.001	0.97	0.23–4.13	0.96
Adjusted SBP CV model^*∗*^	7.07	2.14–23.4	0.001	0.80	0.17–3.88	0.79

Cohort	Persistent proximal vessel occlusion (*n* = 46)			No persistent proximal vessel occlusion (*n* = 140)		

SBP CV	18.1	2.22–148	0.01	3.07	1.11–8.49	0.03
Adjusted SBP CV model^*∗*^	48.9	4.08–587	0.002	1.93	0.67–5.61	0.23

^*∗*^Adjusted for admission NIHSS, patient age, endovascular therapy, IV tPA administration, and premorbid mRS.

**Table 5:** Unadjusted and adjusted ordinal logistic regression models fitted to outcome of mRS with main predictor variable of SBP CV, with models stratified by terciles of mean SBP.

Variable	Unadjusted OR for 1-point mRS shift	95% CI	*p* value	Adjusted OR for 1-point mRS shift^*∗*^	95% CI	*p* value
0–24 hours (*n* = 215)						

SBP CV, lowest SBP tercile	3.76	1.55–9.15	0.01	4.05	1.43–11.5	0.001
(mean SBP 93–123 mmHg)						
SBP CV in middle tercile	1.73	0.55–5.46	0.35	0.58	0.15–2.27	0.44
(mean SBP 124–139 mmHg)						
SBP CV in highest tercile	1.26	0.46–3.39	0.66	1.41	0.45–4.41	0.56
(mean SBP 140–191 mmHg)						

0–72 hours (*n* = 202)						

SBP CV in lowest tercile	8.05	2.50–25.9	<0.001	6.39	1.63–25.1	0.003
(mean SBP 84–123 mmHg)						
SBP CV in middle tercile	2.61	0.75–9.07	0.13	0.68	0.13–3.72	0.66
(mean SBP 124–138 mmHg)						
SBP CV in highest tercile	0.78	0.21–2.89	0.71	1.25	0.31–4.99	0.75
(mean SBP 139–192 mmHg)						

0–120 hours (*n* = 186)						

SBP CV in lowest tercile	10.5	2.25–48.6	0.002	8.77	1.67–46.2	0.01
(mean SBP 93–123 mmHg)						
SBP CV in middle tercile	1.81	0.49–6.65	0.37	1.06	0.23–4.88	0.94
(mean SBP 124–139 mmHg)						
SBP CV in highest tercile	1.56	0.30–8.01	0.59	1.47	0.29–7.49	0.64
(mean SBP 140–191 mmHg)						

^*∗*^Adjusted for admission NIHSS, patient age, admission glucose, PPVO, and premorbid mRS.
